# Promoting Healthy Aging Through Mindfulness and Yoga: A Systematic Review of Interventions for People Living with HIV Who Use Drugs or Who Have a History of Substance Use

**DOI:** 10.3390/ijerph22111685

**Published:** 2025-11-06

**Authors:** Chase M. Bryer, Garrett S. Stang, Alexandra B. Collins, Laura N. Haygood, Tria Blu Wakpa, Jeffrey Proulx

**Affiliations:** 1 Department of Behavioral and Social Sciences, Brown University, Providence, RI 02912, USA; garrett_stang@brown.edu (G.S.S.); jeffrey_proulx@brown.edu (J.P.); 2 Department of Community Health, Tufts University, Medford, MA 02155, USA; alexandra_b.collins@tufts.edu; 3 Brown University Library, Brown University, Providence, RI 02912, USA; laura_haygood@brown.edu; 4 Department of World Arts and Cultures/Dance, University of California, Los Angeles, CA 90095, USA; triabluwakpa@g.ucla.edu

**Keywords:** HIV, substance use, healthy aging, mindfulness, yoga

## Abstract

Advances in HIV care and broader access to antiretroviral therapy (ART) have extended life expectancy for people living with HIV, yet aging-related health concerns, especially among those who use substances, remain under-researched. This systematic review examined the effects of mindfulness and yoga-based interventions on healthy aging outcomes in adults living with HIV who use drugs or who have a history of substance use. We searched Medline, Embase, PsycINFO, and Scopus for randomized controlled trials, cohort studies, quasi-experimental designs, and pre–post intervention studies that assessed healthy aging indicators—broadly defined to include physical, cognitive, and mental health outcomes—along with substance use and HIV-related quality of life measures such as ART adherence. All five studies that met inclusion criteria found mindfulness and yoga interventions to be feasible and acceptable for people living with HIV who use substances, with some evidence suggesting reductions in stress and substance use, as well as improvements in ART adherence. However, most studies were small, short-term pilot trials, none focused specifically on older adults, and few used HIV-specific measures of quality of life or healthy aging. Notably, older adults living with HIV were underrepresented in the studies examined, pointing to the need for increased engagement with older adults living with HIV. These findings suggest promise for mindfulness and yoga for promoting healthy aging among people living with HIV who use substances. Future research should prioritize larger sample sizes and longitudinal designs to better understand clinical efficacy. Existing interventions may be enhanced by incorporating multilevel, community-driven approaches that reflect lived experiences and expand definitions of aging well among people living with HIV who use drugs or who have a history of substance use.

## 1. Introduction

With access to care and antiretroviral therapy (ART), people living with HIV now have life expectancies comparable to those without HIV [1,2]. As a result, the global population of people living with HIV is aging, with an increasing number reaching age 50 and beyond. In the United States, it is estimated that by 2030, 70% of individuals living with HIV will be over the age of 50 [3]. However, people living with HIV have a higher prevalence of substance use, polysubstance use (use of more than one substance), and substance use disorder (SUD) diagnoses compared to the general public [4,5,6]. This can significantly impact life expectancy and contribute to adverse health outcomes across the lifespan [7].

Globally, an estimated 50% of people living with HIV report lifetime or current substance use, and in the United States, about 11% meet criteria for a substance use disorder [8,9]. Injection drug use also accounts for roughly 10% of all new HIV infections worldwide [10]. In 2022, people who inject drugs were estimated to experience 14 times higher risk of acquiring HIV compared to the general adult population [11]. As people age with HIV, they face elevated risks for opportunistic infections, HIV-associated comorbidities, accelerated aging, and increased loneliness stemming from HIV-and-aging-related stigma, all of which underscore the importance of coordinated care strategies [11,12,13,14,15]. Despite these realities, the specific aging experiences and needs of people living with HIV who use drugs or have a history of substance use have been largely overlooked.

Traditional notions of “successful aging,” such as Rowe and Kahn’s (1997) definition emphasizing low disease risk, high cognitive and physical function, and active engagement, do not fully capture the lived experiences of those aging with chronic and stigmatized conditions like HIV and SUDs [16,17]. Mainstream aging frameworks must be expanded to reflect the complex realities of people living with HIV, especially those with co-occurring substance use disorders (SUDs). Co-occurring factors such as SUDs and HIV contribute to what has been described as a syndemic burden, as they often co-occur with other health conditions and social stressors like discrimination and racism [18,19].

While individuals with SUDs are more likely to engage in concurrent alcohol and drug use, smoke cigarettes, and report lower quality of life [20,21], these outcomes cannot be understood in isolation from the broader contexts in which they occur [22]. Elevated rates of anxiety and depression [23] intersect with social and structural drivers such as housing instability, targeted policing, HIV stigma, and even legacies of land dispossession, all of which shape health trajectories and aging outcomes [24,25]. Thus, addressing substance use alone is insufficient for promoting healthy aging among people living with HIV, as structural and environmental conditions remain central determinants of wellness across the course of life [26].

Premature and accelerated cognitive aging is well-documented among people living with HIV, particularly among those who use substances [27,28,29,30]. For example, people living with HIV who inject drugs face higher rates of mortality from AIDS, overdose, and liver disease—primarily due to social–structural factors like criminalization and stigma—but survival rates improve when medications for opioid use disorder and ART are accessible [31,32,33,34,35]. Additionally, people living with HIV who smoke cigarettes lose an estimated 13 years of life due to smoking, compared to only five years due to HIV infection itself [36].

Mindfulness and yoga encompass diverse practices that take many forms, ranging from breathwork and meditation to physical postures and mindful movement. In 1977, Jon Kabat-Zinn developed one of the first programs, Mindfulness-Based Stress Reduction (MBSR), in a Western clinical setting aimed at using mindfulness to address stress-related disorders in chronically ill patients. Standard MBSR is an eight-week program featuring practices like body scans, mindful breathing, sitting meditation, and informal mindfulness to help individuals cultivate mindful attention for coping with stress, pain, and chronic conditions. MBSR integrates Buddhist meditation, other contemplative practices such as yoga, and modern psychological theories of stress and coping. This foundational work produced a secular, mainstream approach to mindfulness and yoga interventions that have been used to address an array of behavior change elements including substance use disorders, chronic disease self-management, violence, suicide/self-injury, and other risk behaviors [37].

Mindfulness- and yoga-based interventions have emerged as promising non-pharmacological strategies to support healthy aging and improve outcomes across the HIV care continuum and may be particularly useful for people living with HIV who use or have used substances [38,39]. Mindfulness and yoga-based approaches have been associated with reductions in depression and improvements in overall quality of life [40,41,42,43]. Additionally, mindfulness-based stress reduction has been identified as a potential key facilitator of symptom management among people living with HIV [43]. Despite these findings, there remains limited understanding of how these interventions specifically impact substance use patterns and healthy aging among people living with HIV who use drugs or have a history of substance use. Given this gap, yoga and mindfulness-based interventions may offer a promising avenue for addressing SUD and promoting healthy aging in this community. This systematic review is guided by the following research question: How effective are yoga and/or mindfulness interventions in addressing healthy aging outcomes among people living with HIV who use drugs or who have a history of substance use? In the studies reviewed, these practices are operationalized in varied ways, including structured yoga sequences, guided breathing exercises, body scans, and awareness-based techniques, and span international contexts.

## 2. Methods

### 2.1. Search Strategy

This systematic review was conducted following the methodology described in the Cochrane Handbook of Systematic Reviews of Interventions [44] and is reported in accordance with the Preferred Reporting Items for Systematic Reviews and Meta-Analyses (PRISMA) guidelines [45]. The review was also pre-registered via OSF’s national Generalized Systematic Review Registration (DOI: 10.17605/OSF.IO/X4CHJ) [46]. A comprehensive search was developed in Ovid Medline by the research librarian (LH), utilizing key papers already identified on the topic. The search utilized MeSH terms and keywords based on the following concepts: HIV, substance use, and yoga/mindfulness. See Supplemental File for our full search strategy, including key words, dates run, and databases used. This search was peer reviewed by another research librarian using the Peer Review of Electronic Search Strategies (PRESS) checklist [47]. Once finalized, the search was translated and run in Embase, Scopus, and APA PsycINFO.

### 2.2. Selection Criteria

The PICOS framework was used to guide the selection criteria. This review included any peer-reviewed evidence on yoga and/or mindfulness-based interventions to address healthy aging among people living with HIV who use drugs or have a history of substance use or used substances, delivered to adults living with HIV (age ≥ 18). Given the dearth of existing literature on healthy aging among people living with HIV, our definition of “healthy aging” was broad, encompassing factors such as substance use reduction, physical functioning, cognitive health, and mental well-being outcomes [48], with ART adherence also included as a key outcome given its proven role in prolonging life expectancy and improving quality of life among this community [49].

Substance use was defined as any level of current drug use, including but not limited to alcohol, tobacco, cannabis, or other illegal substances (e.g., crack, methamphetamine). Given that even low levels of drug use may impact healthy aging, studies were eligible for inclusion regardless of whether participants had a formal diagnosis or classification of a SUD. Including all substances in this search was necessary to capture a broad spectrum of intervention approaches and outcomes relevant to the unique needs of these communities.

Eligible study designs included randomized controlled trials, cohort studies, quasi-experimental studies, and pre–post intervention studies. Qualitative studies were excluded to maintain methodological consistency in exploring outcomes across studies. Additionally, a thorough synthesis of qualitative data would require substantial time and resources to do justice to the richness of these findings, which was beyond the timeline of the current review. Eligible comparison groups included placebo, standard care, alternative treatments, non-tailored treatment-related comparisons, or no comparison at all, ensuring the capture of a wide range of studies. Substance use outcomes included cessation or reductions in use. Primary cessation or reduction outcomes included abstinence measures, likely 7- or 30-day point prevalence abstinence, the most commonly used metric among trials. Ineligible studies included non-interventional studies or interventions that did not include a mindfulness- and yoga-based component.

### 2.3. Data Extraction

The results were exported from the databases and uploaded to Covidence for screening and deduplication. Covidence is a web-based collaboration software platform that streamlines the production of systematic and other literature reviews. Following deduplication, a title and abstract screening was conducted, followed by a full-text review by two independent reviewers (CB, GS), with conflicts resolved through discussion. Data was extracted from five articles using an extraction form developed by the reviewers (CB, GS). The extracted data comprised general article information, target substance, intervention, study design, brief participant characteristics, and outcomes measured (including brief interpretation). The extracted data was narratively synthesized into a summary table (Table 1). Narrative synthesis was then used to integrate and interpret findings from multiple studies.

### 2.4. Quality Assessment

Each study was independently appraised for risk of bias by two reviewers (CB, GS) using Quality Assessment of Controlled Intervention Studies (for 4 RCTs) and ROBINS-I tool (Risk of Bias in Non-randomized Studies—Interventions) (for 1 intervention study with no randomization). Each tool uses a comprehensive set of questions that assesses potential biases and methodological rigor of studies. CB and GS screened each study individually and discussed any discrepancies to critically appraise each study as “Good”, “Fair” or “Poor”. Studies were rated as “Good” if the methods adequately minimized bias across all domains, “Fair” if bias was adequately minimized in all but two domains, and “Poor” if three or more domains had substantial concerns likely to affect the validity of the findings. Any disagreements between reviewers during study selection and data extraction were resolved through discussion and consensus, with a third reviewer consulted if discrepancies remained. Quality ratings of Good, Fair, or Poor were determined based on criteria outlined in the respective checklists: for RCTs, domains such as randomization, allocation concealment, blinding, completeness of outcome data, and selective reporting were considered; for non-randomized studies, ratings were based on bias due to confounding, participant selection, intervention classification, deviations from intended interventions, missing data, outcome measurement, and selective reporting.

## 3. Results

### 3.1. Results of the Literature Search

**Figure 1:**
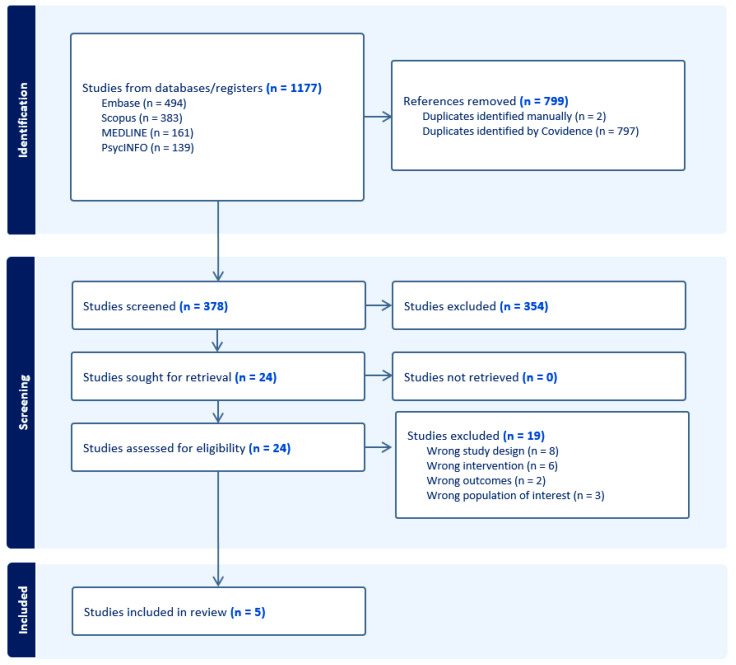
PRISMA flow diagram of studies related to mindfulness and/or yoga among people living with HIV who use drugs or have a history of substance use.

The results of the study search and screening from the database are shown in Figure 1. A total of five studies were identified that evaluated mindfulness or yoga-based interventions delivered to people living with HIV who use drugs or who have a history of substance use (see Table 1 for an overview of included studies and study characteristics). Across these studies, feasibility and acceptability were consistently reported as high, with several demonstrating preliminary effectiveness in improving ART adherence, reducing substance use, and mitigating psychosocial distress. The average number of participants across studies was approximately 50.

### 3.2. Risk of Bias Estimation

Four studies were randomized controlled trials [50,51,52,53] and one was a non-randomized intervention trial [54]. CB and GS independently rated each study using the Quality Assessment of Controlled Intervention Studies checklist from the National Heart, Lung, and Blood Institute for RCTs, and the ROBINS-I Risk of Bias Assessment for the non-randomized study. CS and CB then compared ratings and resolved discrepancies through discussion. On a scale of Good, Fair, or Poor, all studies were rated as “Good” in terms of minimizing bias. See Section 2.4 for specifics outlining our quality appraisal process.

**Table 1:** Characteristics of included studies.

Author	Substance	Sample	Study Design/Setting	Intervention	Substance Use Outcome	Healthy Aging Outcome	Interpretation
Agarwal et al. (2015) [53]	Crack cocaine	N = 24	RCT; assessed at baseline, 2 and 4 months in Miami	Yoga/meditation	Not reported	↓ PSS post-intervention vs. baseline (MD = 4.7, *p* < 0.05); ↓ IES intrusion at post (MD = 7.8, *p* < 0.05) & follow-up (MD = 5.4, *p* < 0.05)	Reduction in perceived stress and trauma-related symptoms
Cioe et al. (2023) [54]	Cigarettes	N = 16	8-week single-group pilot	30-day Unwinding Anxiety app	↑ Readiness to quit at week 4 (b = 0.56, *p* = 0.002), not significant at week 8 (b = 0.34, *p* = 0.30)	↓ Anxiety at week 4 (b = −5.5, *p* = 0.004) & week 8 (b = −5.1, *p* = 0.008)	Improved anxiety symptoms, slight gain in readiness to quit
Luoma et al. (2023) [50]	Injection drugs	N = 100	RCT at harm reduction NGO in Russia	ACT-based stigma reduction group	No group differences in drug use at 1 mo (MD = 0.00, *p* = 1.0) or 6 mo (MD = −3.33, *p* = 0.210); ↑ ART initiation (PD = 0.17, *p* = 0.005)	No difference in HIV stigma (AMD = 0.40, *p* = 0.14); ↑ treatment use (PD = 0.17–0.21, *p* = 0.008–.017)	No change in drug use; improved linkage to HIV and substance use treatment
Magidson et al. (2021) [51]	Alcohol & other drugs	N = 61	Hybrid RCT in South Africa	Khanya (peer-delivered behavioral intervention)	↓ PEth (t = 4.16, *p* = 0.01); ↓ high-risk WHO-ASSIST category 30–40 pts	↑ ART adherence (t = +6.4% Khanya vs. −22.3% ETAU, *p* < 0.05); adherence linked to suppression (t = 2.31, *p* = 0.02)	Reduced substance use risk; improved ART adherence
Wimberly et al. (2018) [52]	Substances (various)	N = 73	RCT; reentry program clients	12-session Hatha yoga	↓ Substance use in yoga group vs. TAU at 1–3 mo (χ^2^ = 11.13, *p* < 0.001)	↓ Stress at 3 mo in yoga group (F = 9.24, *p* < 0.05)	Yoga reduced stress and substance use in returning citizens

Acronyms Key: PSS: Perceived Stress Scale; IES: Impact of Event Scale; Peth: Phosphatidylethanol (an alcohol biomarker); WHO-ASSIST: World Health Organization Alcohol, Smoking and Substance Involvement Screening Test; ETAU: enhanced treatment as usual. Arrows used to visually indicate directionality of change or association in presented outcomes.

### 3.3. Study Characteristics

Basic information on the inclusion of all studies is shown in Table 1. Agarwal et al. (2015) piloted a yoga/mindfulness intervention among people living with HIV who use crack cocaine, reporting reductions in perceived stress and trauma-related symptoms and suggesting a need for a larger randomized trial [53]. Participants were on average about 48 years old, and the majority were male (about 63%), with approximately 38% female participants. Similarly, Wimberly et al. (2018) found that a 12-session yoga intervention significantly reduced stress and substance use among returning citizens living with HIV compared to treatment-as-usual [52]. Participants were on average about 45 years old, and the majority were male (about 69%), followed by female (29%), and transgender (3%). Magidson et al. (2021) evaluated Khanya, a peer-delivered intervention in South Africa combining behavioral activation and mindfulness components, finding significantly improved ART adherence and reduced alcohol use compared to enhanced TAU [51]. Participants were on average about 37 years old, and just over half were female (about 54%), with 46% male participants. Luoma et al. (2023) tested a stigma-focused mindfulness-based intervention among people living with HIV with injection drug use in Russia, finding greater initiation of ART and substance use care in the intervention group, though reductions in stigma scores and drug use frequency were not statistically significant [50]. The average age of participants was 38, with a near 50% split between male and female participants.

Other studies focused on mobile mindfulness applications. Cioe et al. (2023) evaluated an app-based mindfulness program (Unwinding Anxiety) for people living with HIV with high baseline anxiety and found significant reductions in anxiety and increased readiness to quit smoking by week four, though this was not sustained at week eight [54]. Participants were on average about 52 years old, and 60% were male, with 40% female participants.

Collectively, these interventions—ranging from in-person yoga to app-based and spiritually infused modalities—suggest that mindfulness and yoga-based interventions may be promising, adaptable strategies for addressing intersecting challenges of HIV and substance use, thereby promoting healthy aging.

## 4. Discussion

This systematic review synthesized evidence from five studies examining mindfulness- and yoga-based interventions delivered to people living with HIV who use drugs or have a history of substance use, with a focus on healthy aging outcomes. All studies were rated ‘Good’ for minimizing bias using validated systematic review measures. Across studies, mindfulness- and yoga-based interventions consistently demonstrated positive effects in supporting healthy aging among people living with HIV who use substances. Taken together, the findings highlight the promise of mindfulness and yoga approaches as feasible and useful strategies to enhance health and wellness for people aging with HIV in the context of substance use. Future research should also address potential co-intervention bias, examining whether participants in different study arms received additional interventions that could have affected outcomes. Across diverse geographic contexts and delivery formats—including in-person, peer-led and app-based—interventions were consistently rated as feasible and acceptable. Several studies reported promising outcomes, including reductions in stress, anxiety, and substance use, as well as improved ART adherence and readiness to quit. These findings illustrate how non-pharmacological approaches, such as mindfulness- and yoga-based interventions, can serve as accessible and culturally adaptable strategies to promote healthy aging and improve quality of life and care engagement among people living with HIV, aligning with existing literature highlighting the possible benefits of yoga and mindfulness for supporting healthy aging [55].

Despite these promising findings, the concept of healthy aging remains underdeveloped in this literature. None of the included studies explicitly defined or measured healthy aging, nor did any target individuals aged 50 or older—a significant limitation given that by 2030, over 70% of people living with HIV in the U.S. will be aged 50 and older [3]. Moreover, no study employed HIV-specific quality of life measures, despite longstanding concerns about their inconsistent use in intervention research [56]. This underscores the need to develop age-relevant, HIV-specific outcome measures that can better capture the multifaceted experiences of older people living with HIV, especially those managing co-occurring substance use and psychosocial stressors. Rosenfeld et al. (2021) suggest that researchers should treat the process of evaluating quality of life as meaningful data itself—using open, narrative, and biographical approaches rather than fixed measurements—to capture the ongoing, contextual, and interpretive nature of how people experience and assess their lives [57]. Rowe and Kahn’s (1997) influential model of successful aging emphasized three domains: minimizing disease and disability, maintaining physical and cognitive function, and sustaining active engagement with life [16]. While this framework advanced aging research, it has been critiqued for overlooking the structural, cultural, and identity-based dimensions of aging, particularly for historically marginalized groups such as non-white, non-heterosexual people living with HIV [58]. Expanding Rowe and Kahn’s (1997) [16] model to include social determinants of health, resilience, stigma navigation, and cultural strengths is critical for capturing a fuller picture of healthy aging across diverse communities, including LGBTQ+ people living with HIV, who experience higher rates of HIV and mental health burden compared to their non-LGBTQ+ counterparts [59]. Fabbre et al. (2025) offer principles for advancing rigor and justice in work with LGBTQ+ aging communities such as deepening visibility and awareness, applying critical perspectives, and engaging in reflexivity, to name a few [60].

For older people living with HIV, healthy aging often involves managing multimorbidity and navigating stigma while simultaneously cultivating resilience, social connectedness, and identity affirmation [17]. For example, maintaining community ties, spiritual engagement, and a sense of purpose can be just as central to perceptions of healthy aging as biomedical indicators like viral suppression or absence of comorbidities [17,61,62]. In this context, mindfulness- and yoga-based interventions may serve not only as tools for stress reduction but also as vehicles for reinforcing agency, reducing internalized stigma, and fostering meaningful connections—all key dimensions of what “healthy aging” looks like for people living with HIV. Integrating these broader psychosocial and cultural domains into definitions of healthy aging would ensure that intervention research reflects the lived experiences and priorities of older people living with HIV, rather than narrowly focusing on disease management. This is particularly important for people living with HIV who use drugs or have a history of substance use, as they may experience distinctive aging-related concerns that warrant further investigation to better understand associated comorbidities. For example, reduced cognitive function has been identified as a significant predictor of mortality among people living with HIV and those with a history of injection drug use [63].

The lack of aging-specific HIV research reflects broader patterns in the literature, in which the distinct needs of Black, Indigenous, and other People of Color communities aging with HIV have often been overlooked [64,65,66]. This gap is particularly urgent given that Indigenous peoples globally experience HIV prevalence rates nearly three times higher than the general population [67]. Numerous barriers exist in HIV care with Indigenous peoples, including stigmatization, linguistic barriers, and mistrust due to legacies of historical trauma [68]. People aging with HIV in rural settings also face challenges including barriers to maintaining adherence [69]. Additionally, LGBTQ+ individuals aging with HIV often encounter layered stigma and structural neglect, with few interventions tailored to their lived experiences [70,71]. People living with HIV in low- and middle-income countries also experience elevated prevalence of comorbidities, illustrating the need for tailored interventions across global contexts [72]. While some studies in this review demonstrated sensitivity to intersectional stigma and community-anchored care approaches, fewer than half reported specific engagement with people living with HIV in the development or delivery of the interventions.

## 5. Limitations

This systematic review offers important insights but is not without limitations. Most of the included studies were pilot trials with small sample sizes and limited statistical power, focusing primarily on feasibility and acceptability rather than clinical efficacy. Moreover, older adults living with HIV were underrepresented across the studies, with the average age of participants remaining below 50, apart from the Cioe et al. study (2023) [54]. Only a few studies employed RCT designs, and even those often lacked long-term follow-up or robust comparison groups. The absence of standardized outcome measures—particularly those capturing healthy aging (e.g., longitudinal) or HIV-specific quality of life—further limits comparability across studies. As such, there is a critical need for larger, methodologically rigorous RCTs that assess intervention effectiveness over time and include diverse communities, particularly older people living with HIV.

Moreover, this systematic review was designed to be expansive and therefore captured studies that ranged from legal to illegal substances. It could be useful to look more specifically at certain substances in the future (e.g., cigarettes vs. injection drug use may render distinctive different outcomes). Relevant interventions that did not explicitly mention mindfulness or yoga in the title/abstract or keywords may have also been excluded. This systematic review also did not include studies using terms such as ‘recovery,’ which may capture relevant literature. Future reviews should consider incorporating such terms to broaden the scope of included studies.

## 6. Conclusions

Although global efforts have reduced new HIV infections, more people are living longer with HIV, reflecting the resilience of a growing community of long-term survivors. This highlights the need for interventions that support healthy aging, especially for those who use drugs or have a history of substance use. These findings suggest mindfulness- and yoga-based interventions show promise for promoting healthy aging, but larger studies with longer follow-up are needed. Future research must meaningfully engage people living with HIV who use drugs or have a history of substance use in intervention design to ensure ethical, effective care [73], particularly for older adults facing age-related challenges and intersectional stigma [74]. HIV-specific quality of life and healthy aging measures are also essential, along with community-anchored definitions of what it means to age well with HIV. Behavioral individual-level interventions alone are insufficient [22,75]. Interventions must encompass integrated multidisciplinary approaches that move beyond viral suppression to address broader health and social needs, including access to drug treatment, harm reduction services, decriminalization, and stable housing [76,77,78,79,80]. Multilevel strategies are needed to address the root causes of drug use and substance use patterns to support the collective agency of people living with HIV, particularly as they age.

## Data Availability

This systematic review was pre-registered with the Open Science Framework (OSF) under the Generalized Systematic Review Registration, created and registered on 24 August 2025. The registration is publicly available at https://archive.org/details/osf-registrations-x4chj-v1 (accessed on 1 November 2025) and can be accessed via DOI: 10.17605/OSF.IO/X4CHJ. No new data were created or analyzed in this study; all data used are from previously published sources cited within the manuscript.

## Supplementary Materials

The following supporting information can be downloaded at: https://www.mdpi.com/article/10.3390/ijerph22111685/s1.
