# Supplementary material for: Promoting Healthy Aging Through Mindfulness and Yoga: A Systematic Review of Interventions for People Living with HIV Who Use Drugs or Who Have a History of Substance Use
Source: Int J Environ Res Public Health. 2025 Nov 6;22(11):1685. doi: 10.3390/ijerph22111685 (PMC12652410; doi:10.3390/ijerph22111685)
Supplement: Supplementary file 1 [file ijerph-22-01685-s001.zip › ijerph-3852601-supplementary.pdf]

Mindfulness and Yoga Based Interventions for Substance Use Reduction and Healthy Aging Among People Living with HIV Who Use Substances: A Systematic Review

Database	Medline	
Platform	Ovid	
Date of last search	May 14, 2025	
Number of results	85	
Notes	Ovid MEDLINE(R) ALL <1946 to May 13, 2025>	
1	exp HIV/ or exp "HIV infections"/	366104
2	(HIV or HIV+ or HTLV-III infection* or HTLV-III-LAV Infection* or T-Lymphotropic Virus Type III Infection* or "acquired immunodeficiency syndrome*" or "acquired immune deficiency syndrome*" or "human immunodeficiency virus*" or "person* living with AIDS" or "people living with AIDS" or PLHIV or PLWH or PLWHA or "wom?n living with AIDS" or WLHIV or "human T-cell lymphotropic" or "AIDS virus*").ti,ab,kf.	413699
3	1 or 2	479244
4	exp Drug Overdose/ or Drug contamination/ or exp Illicit Drugs/ or Fentanyl/ or Xylazine/ or Naloxone/ or exp Substance-Related Disorders/ or Heroin/ or Narcotics/ or Methamphetamine/ or exp Cocaine/ or exp Benzodiazepines/ or Drug users/ or Alcoholics/ or Alcohol drinking/ or "Tobacco, Smokeless"/ or "Tobacco Use Cessation"/ or exp "Tobacco Use"/ or exp "Tobacco Products"/ or "Nicotine"/ or "Electronic Nicotine Delivery Systems"/ or exp Smoking/ or "Smoking Cessation"/ or "Marijuana Smoking"/ or Cannabis/	704337
5	(opioid* or opiate* or heroin or fentanyl or oxycodone or hydrocodone or percocet or ketamine or designer drug* or club drug* or inject* drug or narcotic* or xylazine or benzodiazepine* or OUD or cocaine or crack or stimulant* or meth or methamphetamine or alcohol* or beer* or wine or liquor* or spirits or vape* or vaping or tobacco or nicotine or smoke* or smoking or Tobacco or cigarette* or cigarillo or cigar* or marijuana or cannabis or weed or hash or hashish or spice or THC or addict* or binge* or	2772226

	binging or ((substance or polysubstance or drug or polydrug) adj5 (abuse or "use" or misuse or overuse or dependen* or addict* or problem* or overdos*)) or ("binge?drinking" or "under?age drinking" or intoxicat* or intravenous or ((cannabis or marijuana or hallucinogen* or opioid* or inhalant or narcotic* or analgesic* or psychotropic or stimulant* or amphetamine* or sedative* or benzo* or opiate* or heroin or opium or morphine or codeine or hydrocodone or oxycodone or cocaine or crack or ectasy or meth* or poly?drug) and (addict* or abus* or misus* or disorder* or mis-use or dependen*)))).ti,ab,kf.	
6	4 or 5	2934590
7	yoga/ or exp mindfulness/ or meditation/	14742
8	(yoga or mindfulness or MBCT or "mindfulness-based cognitive therapy" or yogic or meditat* or MBSR or "mindfulness-based stress reduction" or mindful* or mind-body awareness or ayurved* or vinyasa or hatha or raja).ti,ab,kf.	40726
9	7 or 8	41996
10	3 and 6 and 9	85

**Database**	**Embase.com**	
Platform	Elsevier	
Date of last search	May 16, 2025	
Number of results	265	
Notes		
#1	**'human immunodeficiency virus'**/exp OR **'human immunodeficiency virus infection'**/exp	977,158
#2	**hiv**:ti,ab,kw OR **hiv+**:ti,ab,kw OR **'htlv-iii infection*'**:ti,ab,kw OR **'htlv-iii-lav infection*'**:ti,ab,kw OR **'t-lymphotropic virus type iii infection*'**:ti,ab,kw OR **'acquired immunodeficiency syndrome*'**:ti,ab,kw OR **'acquired immune deficiency syndrome*'**:ti,ab,kw OR **'human immunodeficiency virus*'**:ti,ab,kw OR **'person* living with aids'**:ti,ab,kw OR **'people living with aids'**:ti,ab,kw OR **plhiv**:ti,ab,kw OR **plwh**:ti,ab,kw OR **plwha**:ti,ab,kw OR **'wom?n living with aids'**:ti,ab,kw OR **wlhiv**:ti,ab,kw OR **'human t-cell lymphotropic'**:ti,ab,kw OR **'aids virus*'**:ti,ab,kw	535,574
#3	#1 OR #2	1,059,169
#4	**'smokeless tobacco'**/exp OR **'smokeless tobacco'** OR **'smoking cessation'**/exp OR **'smoking cessation'** OR **'tobacco use'**/exp OR **'tobacco use'** OR **'tobacco'**/exp OR **'tobacco'** OR **'nicotine'**/exp OR **'nicotine'** OR **'electronic cigarette'**/exp OR **'electronic cigarette'** OR **'smoking'**/exp OR **'smoking'** OR **'cannabis smoking'**/exp OR **'cannabis smoking'** OR	2,118,457

	**'cannabis'**/exp OR **'cannabis'** OR **'drug overdose'**/exp OR **'drug overdose'** OR **'drug contamination'**/exp OR **'drug contamination'** OR **'illicit drug'**/exp OR **'illicit drug'** OR **'fentanyl'**/exp OR **'fentanyl'** OR **'xylazine'**/exp OR **'xylazine'** OR **'naloxone'**/exp OR **'naloxone'** OR **'drug dependence'**/exp OR **'drug dependence'** OR **'diamorphine'**/exp OR **'diamorphine'** OR **'narcotic agent'**/exp OR **'narcotic agent'** OR **'methamphetamine'**/exp OR **'methamphetamine'** OR **'cocaine'**/exp OR **'cocaine'** OR **'benzodiazepine'**/exp OR **'benzodiazepine'** OR **'drug use'**/exp OR **'drug use'** OR **'alcoholism'**/exp OR **'alcoholism'** OR **'alcohol consumption'**/exp OR **'alcohol consumption'**	
#5	**opioid***:ti,ab,kw OR **opiate***:ti,ab,kw OR **heroin**:ti,ab,kw OR **fentanyl**:ti,ab,kw OR **oxycodone**:ti,ab,kw OR **hydrocodone**:ti,ab,kw OR **percocet**:ti,ab,kw OR **ketamine**:ti,ab,kw OR **'designer drug*'**:ti,ab,kw OR **'club drug*'**:ti,ab,kw OR **'inject* drug'**:ti,ab,kw OR **narcotic***:ti,ab,kw OR **xylazine**:ti,ab,kw OR **benzodiazepine***:ti,ab,kw OR **oud**:ti,ab,kw OR **cocaine**:ti,ab,kw OR **crack**:ti,ab,kw OR **stimulant***:ti,ab,kw OR **meth**:ti,ab,kw OR **methamphetamine**:ti,ab,kw OR **alcohol***:ti,ab,kw OR **beer***:ti,ab,kw OR **wine**:ti,ab,kw OR **liquor***:ti,ab,kw OR **spirits**:ti,ab,kw OR **vape***:ti,ab,kw OR **vaping**:ti,ab,kw OR **nicotine**:ti,ab,kw OR **smoke***:ti,ab,kw OR **smoking**:ti,ab,kw OR **tobacco**:ti,ab,kw OR	4,004,658

	**cigarette***:ti,ab,kw OR **cigarillo**:ti,ab,kw OR **cigar***:ti,ab,kw OR **marijuana**:ti,ab,kw OR **cannabis**:ti,ab,kw OR **weed**:ti,ab,kw OR **hash**:ti,ab,kw OR **hashish**:ti,ab,kw OR **spice**:ti,ab,kw OR **thc**:ti,ab,kw OR **addict***:ti,ab,kw OR **binge***:ti,ab,kw OR **binging**:ti,ab,kw OR (((**substance** OR **polysubstance** OR **drug** OR **polydrug**) NEAR/5 (**abuse** OR **use** OR **misuse** OR **overuse** OR **dependen*** OR **addict*** OR **problem*** OR **overdos***)):ti,ab,kw) OR binge$drinking:ti,ab,kw OR 'under$age drinking':ti,ab,kw OR **intoxicat***:ti,ab,kw OR **intravenous**:ti,ab,kw OR ((**cannabis**:ti,ab,kw OR **marijuana**:ti,ab,kw OR **hallucinogen***:ti,ab,kw OR **opioid***:ti,ab,kw OR **inhalant**:ti,ab,kw OR **narcotic***:ti,ab,kw OR **analgesic***:ti,ab,kw OR **psychotropic**:ti,ab,kw OR **stimulant***:ti,ab,kw OR **amphetamine***:ti,ab,kw OR **sedative***:ti,ab,kw OR **benzo***:ti,ab,kw OR **opiate***:ti,ab,kw OR **heroin**:ti,ab,kw OR **opium**:ti,ab,kw OR **morphine**:ti,ab,kw OR **codeine**:ti,ab,kw OR **hydrocodone**:ti,ab,kw OR **oxycodone**:ti,ab,kw OR **cocaine**:ti,ab,kw OR **crack**:ti,ab,kw OR **ectasy**:ti,ab,kw OR **meth***:ti,ab,kw OR poly$drug:ti,ab,kw) AND (**addict***:ti,ab,kw OR **abus***:ti,ab,kw OR **misus***:ti,ab,kw OR **disorder***:ti,ab,kw OR **'mis use'**:ti,ab,kw OR **dependen***:ti,ab,kw))	
#6	#4 OR #5	4,785,741
#7	**'yoga'**/exp OR **'mindfulness'**/exp OR **'meditation'**/exp	40,142

#8	**yoga**:ti,ab,kw OR **mindfulness**:ti,ab,kw OR **mbct**:ti,ab,kw OR **'mindfulness-based cognitive therapy'**:ti,ab,kw OR **yogic**:ti,ab,kw OR **meditat***:ti,ab,kw OR **mbsr**:ti,ab,kw OR **'mindfulness-based stress reduction'**:ti,ab,kw OR **mindful***:ti,ab,kw OR **'mind-body awareness'**:ti,ab,kw OR **ayurved***:ti,ab,kw OR **vinyasa**:ti,ab,kw OR **hatha**:ti,ab,kw OR **raja**:ti,ab,kw	58,865
#9	#7 OR #8	68,381
#10	#3 AND #6 AND #9	265

**Database**	**Scopus**
Platform	Elsevier
Date of last search	May 16, 2025
Number of results	199
Notes	

(TITLE-ABS-KEY (hiv OR hiv+ OR "HTLV-III infection*" OR "HTLV-III-LAV Infection*" OR "T-Lymphotropic Virus Type III Infection*" OR "acquired immunodeficiency syndrome*" OR "acquired immune deficiency syndrome*" OR "human immunodeficiency virus*" OR "person* living with AIDS" OR "people living with AIDS" OR plhiv OR plwh OR plwha OR "wom*n living with AIDS" OR wlhiv OR "human T-cell lymphotropic" OR "AIDS virus*")) AND (TITLE-ABS-KEY (opioid* OR opiate* OR heroin OR fentanyl OR oxycodone OR hydrocodone OR percocet OR ketamine OR "designer drug*" OR "club drug*" OR "inject* drug" OR narcotic* OR xylazine OR benzodiazepine* OR oud OR cocaine OR crack OR stimulant* OR meth OR methamphetamine OR alcohol* OR beer* OR wine OR liquor* OR spirits OR vape* OR vaping OR tobacco OR nicotine OR smoke* OR smoking OR tobacco OR cigarette* OR cigarillo OR cigar* OR marijuana OR cannabis OR weed OR hash OR hashish OR spice OR thc OR addict* OR binge* OR binging OR ((substance OR polysubstance OR drug OR polydrug) W/5 (abuse OR use OR misuse OR overuse OR dependen* OR addict* OR problem* OR overdos*)) OR (binge*drinking OR "under*age drinking" OR intoxicat* OR intravenous OR ((cannabis OR marijuana OR hallucinogen* OR opioid* OR inhalant OR narcotic* OR analgesic* OR psychotropic OR stimulant* OR amphetamine* OR sedative* OR benzo* OR opiate* OR heroin OR opium OR morphine OR codeine OR hydrocodone OR oxycodone OR cocaine OR crack OR ectasy OR meth* OR poly*drug) AND (addict* OR abus* OR misus* OR disorder* OR mis-use OR dependen*))))) AND (TITLE-ABS-KEY (yoga OR mindfulness OR mbct OR "mindfulness-based cognitive therapy" OR yogic OR meditat* OR mbsr OR "mindfulness-based stress reduction" OR mindful* OR "mind-body awareness" OR ayurved* OR vinyasa OR hatha OR raja))	199

Database	APA PsycInfo	
Platform	EBSCO	
Date of last search	May 16, 2025	
Number of results	70	
Notes		
S1	DE "HIV" OR DE "AIDS"	50,997
S2	((TI HIV OR AB HIV OR SU HIV) OR (TI HIV+ OR AB HIV+ OR SU HIV+) OR (TI "HTLV-III infection*" OR AB "HTLV-III infection*" OR SU "HTLV-III infection*") OR (TI "HTLV-III-LAV Infection*" OR AB "HTLV-III-LAV Infection*" OR SU "HTLV-III-LAV Infection*") OR (TI "T-Lymphotropic Virus Type III Infection*" OR AB "T-Lymphotropic Virus Type III Infection*" OR SU "T-Lymphotropic Virus Type III Infection*") OR (TI "acquired immunodeficiency syndrome*" OR AB "acquired immunodeficiency syndrome*" OR SU "acquired immunodeficiency syndrome*") OR (TI "acquired immune deficiency syndrome*" OR AB "acquired immune deficiency syndrome*" OR SU "acquired immune deficiency syndrome*") OR (TI "human immunodeficiency virus*" OR AB "human immunodeficiency virus*" OR SU "human immunodeficiency virus*") OR (TI "person* living with AIDS" OR AB "person* living with AIDS" OR SU "person* living with AIDS") OR (TI "people living with AIDS" OR AB "people living with AIDS" OR SU "people living with AIDS") OR (TI PLHIV OR AB PLHIV OR SU PLHIV) OR (TI PLWH OR AB PLWH OR SU PLWH) OR (TI PLWHA OR AB PLWHA OR SU PLWHA) OR (TI "wom#n living with AIDS" OR AB "wom#n living with AIDS" OR SU "wom#n living with AIDS") OR (TI WLHIV OR AB WLHIV OR SU WLHIV) OR (TI "human T-cell lymphotropic" OR AB "human T-cell lymphotropic" OR SU "human T-cell lymphotropic") OR (TI "AIDS virus*" OR AB "AIDS virus*" OR SU "AIDS virus*"))	66,658
S3	S1 OR S2	68,262
S4	DE "Tobacco Smoking" OR DE "Smoking Cessation" OR DE "Electronic Cigarettes" OR DE "Vaping" OR DE "Tobacco Use Disorder" OR DE "Nicotine" OR ((((((((DE "Drug Overdoses" OR DE "Fentanyl" OR DE "Drugs" OR DE "Drug Dependency") OR (DE "Naloxone")) OR (DE	222,450

	"Substance Use Disorder" OR DE "Drug Addiction" OR DE "Substance Related and Addictive Disorders" OR DE "Opioid Use Disorder")) OR (DE "Heroin" OR DE "Heroin Use Disorder")) OR (DE "Narcotic Drugs")) OR (DE "Methamphetamine")) OR (DE "Cocaine" OR DE "Crack Cocaine")) OR (DE "Benzodiazepines")) OR (DE "Alcohol Use" OR DE "Alcohol Use Disorder" OR DE "Alcohol Intoxication" OR DE "Alcoholism")	
S5	(((TI opioid* OR AB opioid* OR SU opioid*) OR (TI opiate* OR AB opiate* OR SU opiate*) OR (TI heroin OR AB heroin OR SU heroin) OR (TI fentanyl OR AB fentanyl OR SU fentanyl) OR (TI oxycodone OR AB oxycodone OR SU oxycodone) OR (TI hydrocodone OR AB hydrocodone OR SU hydrocodone) OR (TI percocet OR AB percocet OR SU percocet) OR (TI ketamine OR AB ketamine OR SU ketamine) OR (TI "designer drug*" OR AB "designer drug*" OR SU "designer drug*") OR (TI "club drug*" OR AB "club drug*" OR SU "club drug*") OR (TI "inject* drug" OR AB "inject* drug" OR SU "inject* drug") OR (TI narcotic* OR AB narcotic* OR SU narcotic*) OR (TI xylazine OR AB xylazine OR SU xylazine) OR (TI benzodiazepine* OR AB benzodiazepine* OR SU benzodiazepine*) OR (TI OUD OR AB OUD OR SU OUD) OR (TI cocaine OR AB cocaine OR SU cocaine) OR (TI crack OR AB crack OR SU crack) OR (TI stimulant* OR AB stimulant* OR SU stimulant*) OR (TI meth OR AB meth OR SU meth) OR (TI methamphetamine OR AB methamphetamine OR SU methamphetamine) OR (TI alcohol* OR AB alcohol* OR SU alcohol*) OR (TI beer* OR AB beer* OR SU beer*) OR (TI wine OR AB wine OR SU wine) OR (TI liquor* OR AB liquor* OR SU liquor*) OR (TI spirits OR AB spirits OR SU spirits) OR (TI marijuana OR AB marijuana OR SU marijuana) OR (TI cannabis OR AB cannabis OR SU cannabis) OR (TI weed OR AB weed OR SU weed) OR (TI hash OR AB hash OR SU hash) OR (TI hashish OR AB hashish OR SU hashish) OR (TI spice OR AB spice OR SU spice) OR (TI THC OR AB THC OR SU THC) OR (TI addict* OR AB addict* OR SU addict*) OR (TI binge* OR AB binge* OR SU binge*) OR (TI binging OR AB binging OR SU binging) OR (((TI substance OR AB substance OR SU substance) OR (TI polysubstance OR AB polysubstance OR SU	821,721

	polysubstance) OR (TI drug OR AB drug OR SU drug) OR (TI polydrug OR AB polydrug OR SU polydrug)) N5 ((TI abuse OR AB abuse OR SU abuse) OR (TI use OR AB use OR SU use) OR (TI misuse OR AB misuse OR SU misuse) OR (TI overuse OR AB overuse OR SU overuse) OR (TI dependen* OR AB dependen* OR SU dependen*) OR (TI addict* OR AB addict* OR SU addict*) OR (TI problem* OR AB problem* OR SU problem*) OR (TI overdos* OR AB overdos* OR SU overdos*))) OR ((TI binge#drinking OR AB binge#drinking OR SU binge#drinking) OR (TI "under#age drinking" OR AB "under#age drinking" OR SU "under#age drinking") OR (TI intoxicat* OR AB intoxicat* OR SU intoxicat*) OR (TI intravenous OR AB intravenous OR SU intravenous) OR (((TI cannabis OR AB cannabis OR SU cannabis) OR (TI marijuana OR AB marijuana OR SU marijuana) OR (TI hallucinogen* OR AB hallucinogen* OR SU hallucinogen*) OR (TI opioid* OR AB opioid* OR SU opioid*) OR (TI inhalant OR AB inhalant OR SU inhalant) OR (TI narcotic* OR AB narcotic* OR SU narcotic*) OR (TI analgesic* OR AB analgesic* OR SU analgesic*) OR (TI psychotropic OR AB psychotropic OR SU psychotropic) OR (TI stimulant* OR AB stimulant* OR SU stimulant*) OR (TI amphetamine* OR AB amphetamine* OR SU amphetamine*) OR (TI sedative* OR AB sedative* OR SU sedative*) OR (TI benzo* OR AB benzo* OR SU benzo*) OR (TI opiate* OR AB opiate* OR SU opiate*) OR (TI heroin OR AB heroin OR SU heroin) OR (TI opium OR AB opium OR SU opium) OR (TI morphine OR AB morphine OR SU morphine) OR (TI codeine OR AB codeine OR SU codeine) OR (TI hydrocodone OR AB hydrocodone OR SU hydrocodone) OR (TI oxycodone OR AB oxycodone OR SU oxycodone) OR (TI cocaine OR AB cocaine OR SU cocaine) OR (TI crack OR AB crack OR SU crack) OR (TI ectasy OR AB ectasy OR SU ectasy) OR (TI meth* OR AB meth* OR SU meth*) OR (TI poly#drug OR AB poly#drug OR SU poly#drug)) AND ((TI addict* OR AB addict* OR SU addict*) OR (TI abus* OR AB abus* OR SU abus*) OR (TI misus* OR AB misus* OR SU misus*) OR (TI disorder* OR AB disorder* OR SU disorder*) OR (TI mis-use OR AB mis-use OR SU mis-use) OR (TI dependen* OR AB dependen* OR SU dependen*)))))) OR (((TI vape* OR AB vape* OR SU vape*) OR (TI vaping	

	OR AB vaping OR SU vaping) OR (TI tobacco OR AB tobacco OR SU tobacco) OR (TI nicotine OR AB nicotine OR SU nicotine) OR (TI smoke* OR AB smoke* OR SU smoke*) OR (TI smoking OR AB smoking OR SU smoking) OR (TI Tobacco OR AB Tobacco OR SU Tobacco) OR (TI cigarette* OR AB cigarette* OR SU cigarette*) OR (TI cigarillo OR AB cigarillo OR SU cigarillo) OR (TI cigar* OR AB cigar* OR SU cigar*)))	
S6	S4 OR S5	839,890
S7	(DE "Yoga" OR DE "Mindfulness-Based Stress Reduction") AND (DE "Mindfulness" OR DE "Mindfulness-Based Cognitive Therapy" OR DE "Mindfulness Measures" OR DE "Mindfulness-Based Interventions" OR DE "Mindfulness-Based Stress Reduction" OR DE "Mindfulness Meditation" OR DE "Meditation")	962
S8	((TI yoga OR AB yoga OR SU yoga) OR (TI mindfulness OR AB mindfulness OR SU mindfulness) OR (TI MBCT OR AB MBCT OR SU MBCT) OR (TI "mindfulness-based cognitive therapy" OR AB "mindfulness-based cognitive therapy" OR SU "mindfulness-based cognitive therapy") OR (TI yogic OR AB yogic OR SU yogic) OR (TI meditat* OR AB meditat* OR SU meditat*) OR (TI MBSR OR AB MBSR OR SU MBSR) OR (TI "mindfulness-based stress reduction" OR AB "mindfulness-based stress reduction" OR SU "mindfulness-based stress reduction") OR (TI mindful* OR AB mindful* OR SU mindful*) OR (TI "mind-body awareness" OR AB "mind-body awareness" OR SU "mind-body awareness") OR (TI ayurved* OR AB ayurved* OR SU ayurved*) OR (TI vinyasa OR AB vinyasa OR SU vinyasa) OR (TI hatha OR AB hatha OR SU hatha) OR (TI raja OR AB raja OR SU raja))	37680
S9	S7 OR S8	37680
S10	S3 AND S6 AND S9	70
